# Differentiating Benign from Malignant Adrenocortical Tumors by a Single Morphological Parameter—a Clinicopathological Study on 837 Adrenocortical Neoplasias

**DOI:** 10.1007/s13193-020-01205-4

**Published:** 2020-09-05

**Authors:** Martin K. Walz, Klaus A. Metz, Sarah Theurer, Cathrin Myland, Pier F. Alesina, Kurt W. Schmid

**Affiliations:** 1 Department of Surgery and Center of Minimally Invasive Surgery, Kliniken Essen-Mitte, Essen, Germany; 2 Institute of Pathology, University Hospital Essen, University of Duisburg-Essen, Essen, Germany

**Keywords:** Adrenocortical carcinoma, Adrenocortical adenoma, Histopathology, Scoring systems, Tumor necrosis, Tumor fragmentation

## Abstract

The morphological differentiation between benign and malignant adrenocortical tumors is an ongoing problem in diagnostic pathology. In recent decades the complex scoring systems have been widely used to calculate the probability of malignancy in adrenocortical tumors on the basis of a variety of histomorphological parameters. We herewith present a substantially simplified method to diagnose adrenocortical carcinoma by a single histomorphological parameter on a consecutive series of more than 800 adrenocortical tumors. Between January 2000 and May 2019, altogether 2305 adrenalectomies for of all types of diseases were removed, approximately 98% by minimally invasive approaches. After exclusion of pheochromocytomas, adrenal ganglioneuromas, adrenal metastases, Cushing’s disease related specimens, and Conn’s adenomas, the present series finally consisted of 837 adrenocortical tumors. All tumors were analyzed by experienced pathologists of a single institution using standard histopathological methods (Hematoxylin-Eosin and Ki67 stained sections). Clinical and histopathologic data were prospectively collected and retrospectively analyzed. Clinically, 385 patients had 420 functioning tumors (FT), and 417 had non-functioning adrenal tumors (NFT). The mean size of FT was 3.8 ± 1.4 cm (range 0.5–16 cm) and for NFT 4.5 ± 1.6 cm (range 1.5–18 cm). Histomorphologically, 32 adrenal tumors were classified as adrenocortical carcinoma (ACC; 3.8%). In all 32 cases (tumor size 9.1 ± 4.0 cm, range 3–18 cm), confluenting tumor necrosis could be demonstrated. The remaining 805 tumors (control group) completely lacked this highly reproducible single morphological feature. Ki67 levels above 10% were found in 31 of 32 ACCs and never in adrenocortical adenomas (ACA). With a mean follow-up of 8.2 years, 24 out of 32 patients primarily diagnosed as ACC developed distant metastases (75.0%), whereas all patients in the control group remained free of local or distant recurrence. We conclude that a single morphological parameter (confluenting tumor necrosis) is sufficient to predict a poor clinical course in adrenocortical tumors. The histomorphological diagnosis of this parameter is straightforward and highly reproducible.

Adrenocortical carcinoma (ACC) is an ultrarare disease that accounts for far less than 0.1% of all carcinomas. The incidence is around 0.6–1.7 per million [1]. Cure can only be offered by surgery removing the affected adrenal gland and potentially organs, structures, and regional lymph nodes. The prognosis of patients with ACC is limited as the 5-year survival is only 20–30%. The diagnosis of malignancy in adrenocortical tumors may be based on clinical and/or morphological criteria. In cases of regional or distant metastasis, the malignant behavior is evident. Contrarily, in cases without proof of metastases at time of removal, both macro- and histopathologic parameters are used to define the respective lesion as benign or malignant. Tumor size is one criterion as the majority of ACCs are typically larger than 8 cm [2, 3]. Nevertheless, histopathology is known as the essential tool to predict biological behavior. Up to now, the scoring systems by Weiss (1984) and van Slooten (1985) have routinely been used for the definition of ACC [4, 5]. They are based on 9 or 7 histopathological parameters, respectively. Those include atypical nuclei, rate of mitosis, invasion, and necrosis as sign of cancer. As scoring systems per se mean risks of false-positive and false-negative results, they are of limited value. The reproducibility of scoring systems is generally questionable which has also been shown in adrenocortical tumors [6]. Nevertheless, the Weiss score is still regarded as the method of choice for the prediction of the clinical behavior in tumors of the adrenal cortex [7] and has been recommended as standard procedures in currently used guidelines [8]. In contrast, based on more than 800 cases, we herewith present a simplified histopathological method to predict favorable or poor outcome in adrenocortical tumors.

## Patients and Methods

From January 2000 to May 2019, data were collected of all patients operated for adrenalectomy at the Department of Surgery, Kliniken Essen-Mitte, in the setting of a prospective study. Overall, 2305 procedures were performed on 2092 patients, 98.4% by minimally invasive approaches (MIS). All consecutive specimens were histologically investigated in the Institute of Pathology, University Hospital Essen, University of Duisburg-Essen. As the aim of the study is to differentiate between benign and malignant adrenocortical tumors, all medullary tumors (pheochromocytoma, ganglioneuroma) and all adrenals removed in cases of Cushing’s disease (pituitary or ectopic) were excluded. Additionally, all cases of aldosteronomas were not considered as these are practically always benign. Adrenal metastases were excluded as well as a variety of other rare indications for adrenalectomy such as in adrenal cysts, angiosarcoma, leiomyosarcoma, and recurrent adrenocortical cancer or for adrenogenital syndrome. Eventually, 837 adrenal tumors met the inclusion criteria. The surgical technique for tumor removal has been described before. In the majority of operations, the posterior retroperitoneoscopic approach—developed by us in 1994 [9]—was used. Few tumors were removed by the transabdominal laparoscopic or an open approach. In minimally invasive cases, adrenal neoplasias were retrieved in a plastic bag. For tumors larger than skin incisions, tissue was routinely fragmentized in a bag—as described earlier [10]. Thereby widening of the surgical incisions and associated morbidity could be avoided. Immediately after removal, all specimens were fixed in 4% buffered formalin and sent for pathologic evaluation. That included gross inspection of the specimen, selection of regions of interest for histologic investigation, dehydration, embedding in paraffin, and conventional staining with Hematoxylin-Eosin. Ki67 proliferation index (MIB-1) and positivity for synaptophysin were routinely determined. Only in very few cases the investigation included antibodies against melan-A, calretinin, alpha-inhibin, and chromogranin A. The study was approved by the ethical board of the University Hospital Essen.

Age, gender, tumor size and side as well as hormonal function were assessed in all patients. Histopathologic diagnosis based on findings of tumor cell necrosis, local infiltration, and distant metastasis was recorded. To obtain long-term results, patients, their general practitioners and endocrinologists were contacted by phone or the patients were personally seen for clinical follow-up. Data were presented as mean values ± standard deviation (with range). For group comparison Fisher’s exact test was used. Significance was accepted for *p* < 0.05. Statistical analyses were performed by a program for personal computers (Prism 6.0; GraphPad, La Lolla, USA).

## Results

The retroperitoneoscopic route for adrenalectomy was used in 803 (95.9%) patients; the laparoscopic transperitoneal access has been chosen in 25 (3.0%) and a laparotomy in 9 (1.1%) patients. Clinically, 385 patients (59 males and 326 females; age 51.2 ± 14.4 years) had 420 functioning tumors (FT; 397 hypercortisolism, 18 virilizing, 1 feminizing, 4 combined secretion) and 417 patients (159 males, 268 females; age 58.8 ± 11.7 years) had 417 non-functioning adrenal tumors (NFT). In the FT group, 35 patients with Cushing’s syndrome had bilateral (*n* = 34) or recurrent unilateral (*n* = 1) neoplasias. Of these 34 patients with bilateral tumors, 26 patients suffered from macronodular hyperplasia, one from micronodular hyperplasia, and 5 from bilateral adenomas. Surgeries were synchronous in 30 patients and metachronous in 4 cases. The mean size of FT was 3.8 ± 1.4 cm (range 0.5–16 cm) and for NFT 4.5 ± 1.6 cm (range 1.5–18 cm).

**Fig. 1:**
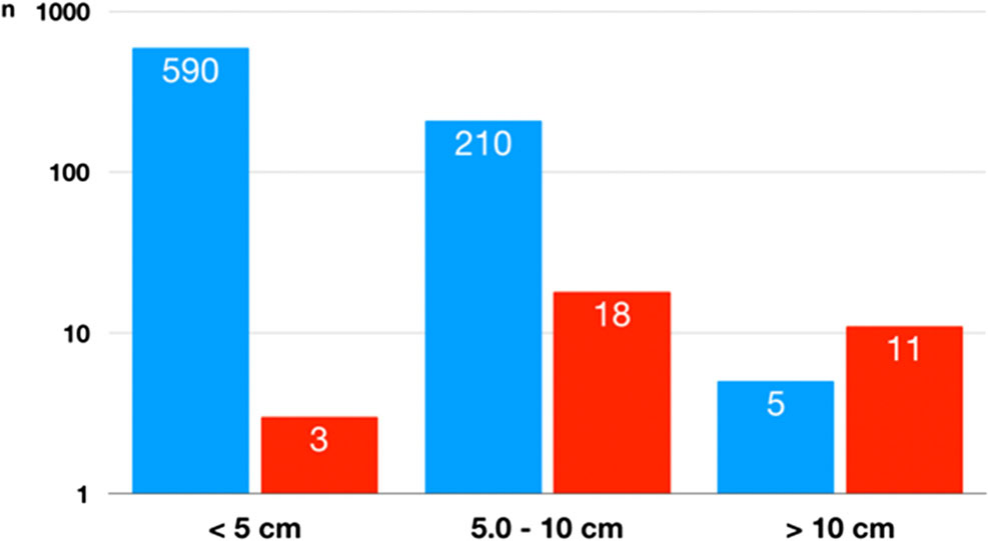
Size of 837 adrenocortical tumors (805 benign, 32 malignant). Benign tumors ( ) and malignant tumors ( ). Logarithmic scale (Essen (2000–2019))

Histomorphologically, in 32 patients (14 males and 18 females; age 51.6 ± 20.0 years), the adrenal tumors were classified as primary adrenocortical carcinoma (ACC; 3.2%). Clinically, 16 of these patients suffered from functioning tumors (10 hypercortisolism, 3 virilizing, 3 mixed secretion). Mean tumor size was 9.1 ± 4.0 cm with a range of 3–18 cm. Three tumors were smaller than 5 cm in diameter, 18 lesions between 5 and 10 cm, and 11 cancers larger than 10 cm. Compared with the benign adrenal tumors, the size was significantly different between both groups (Fig. 1; *p* < 0.001). Included are two children (age 1.6 and 3.6 years, respectively). Both had virilizing tumors (4 cm and 15 cm of diameter). All 73 specimens with macronodular hyperplasia (68 functioning, 5 non-functioning) were histologically classified as benign.

**Fig. 2:**
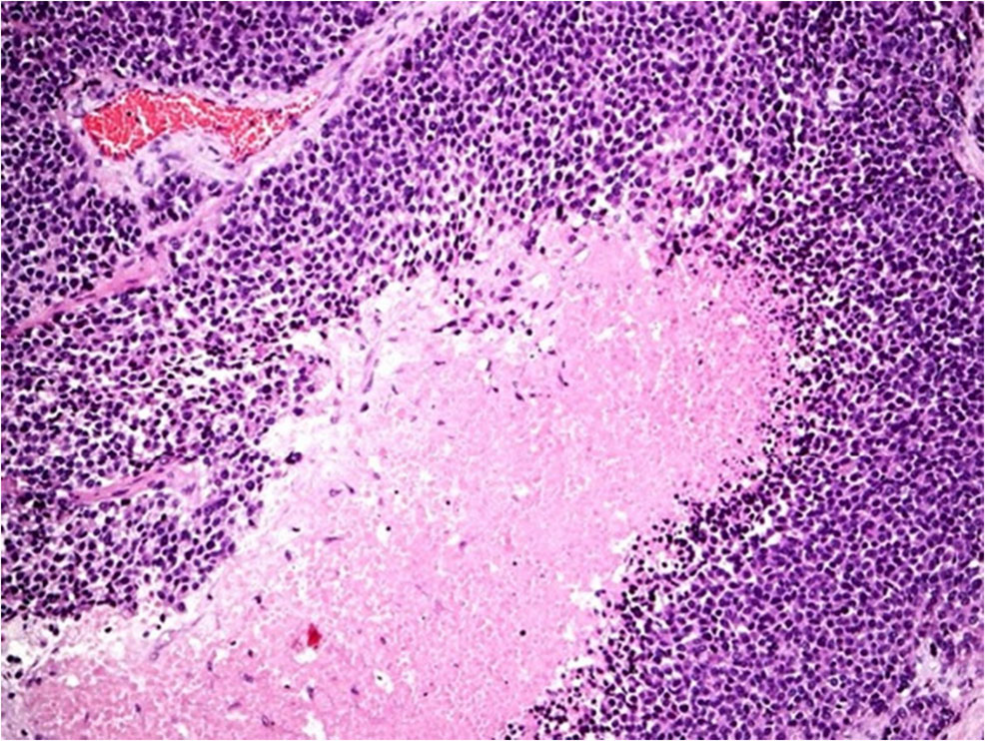
Histopathology (Hematoxylin-Eosin (× 100)), confluenting tumor necrosis in adrenocortical cancer. Typical finding in all 32 adrenal tumors diagnosed as adrenocortical cancer

**Fig. 3:**
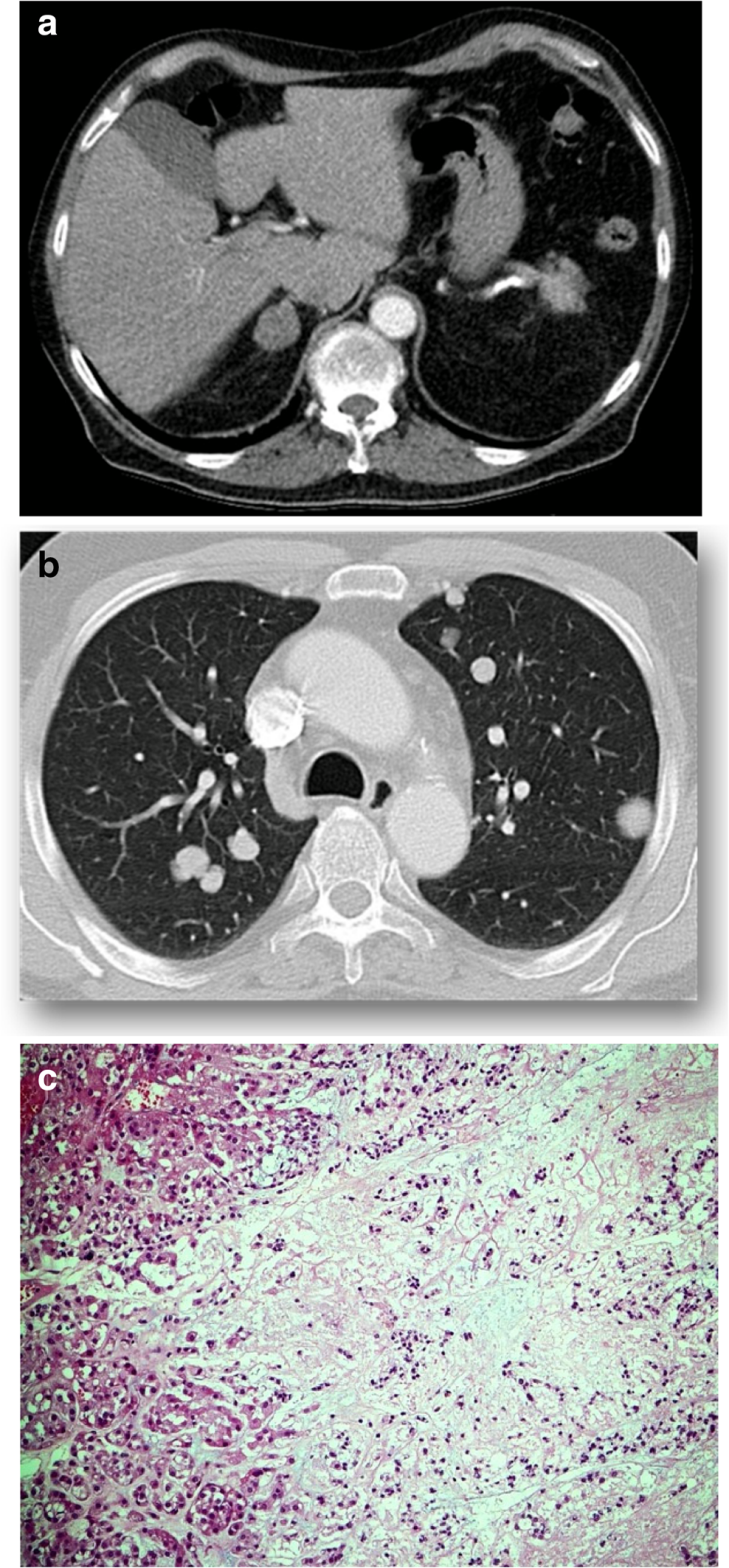
Male patient (71 years). (**a**) Computed tomography showing a 3-cm right-sided adrenal tumor. (**b**) Adrenocortical cancer proven by lung metastasis. (**c**) Histopathology showing the area of necrosis in the carcinoma. Hematoxylin-Eosin (× 100)

**Fig. 4:**
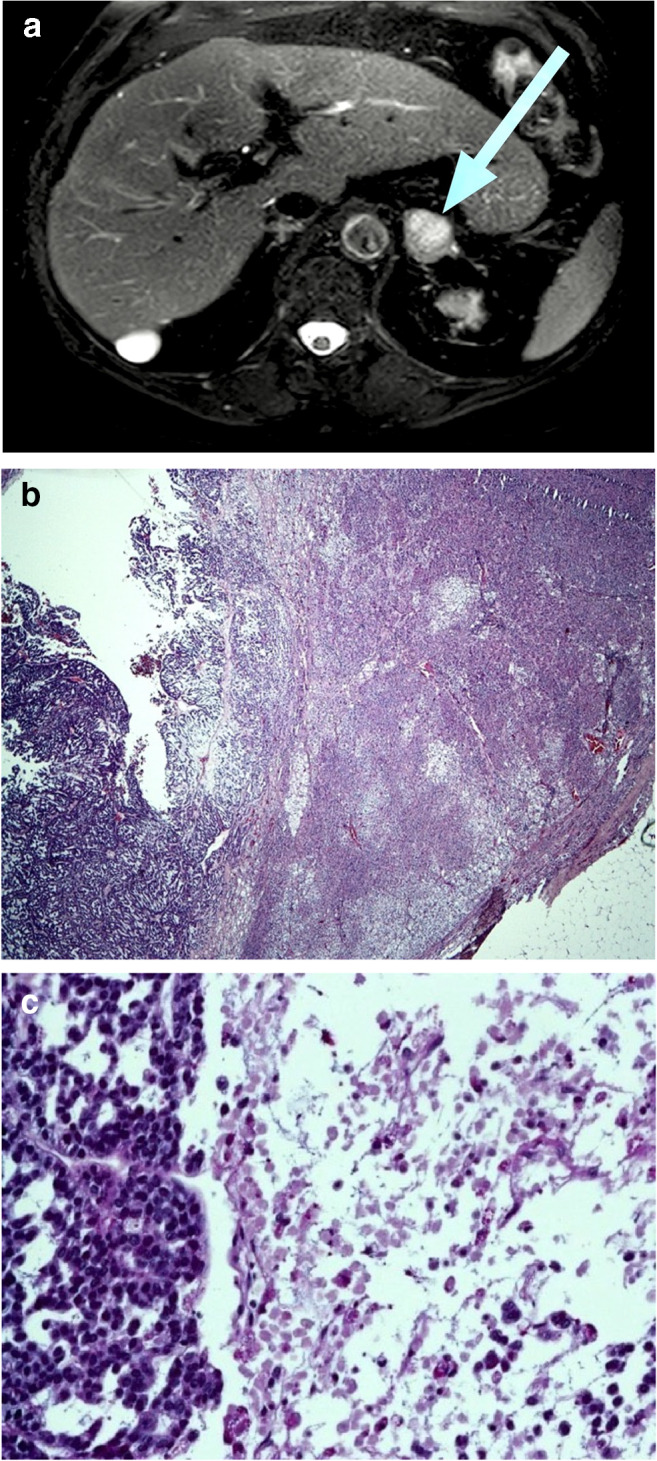
Female patient (82 years). (**a**) Magnetic resonance imaging (T2 weighted) showing a 3-cm left-sided adrenal tumor. (**b**) Histopathology (Hematoxylin-Eosin (× 2)). Adrenocortical carcinoma (left side), arising from an adenoma (right side). (**c**) Histopathology (Hematoxylin-Eosin (× 200)). Adrenocortical carcinoma (potentially arising from an adenoma), typical necrosis (right side)

**Fig. 5:**
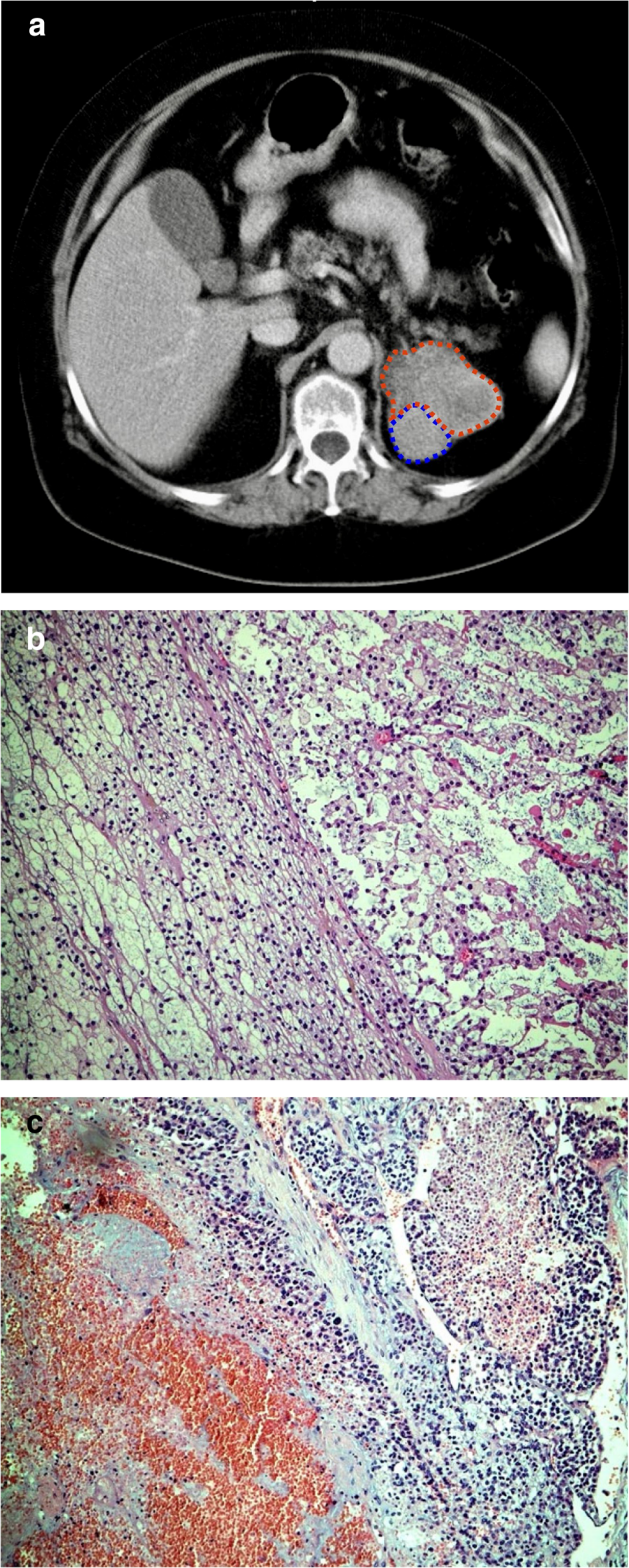
Female patient (64 years). (**a**) Computed tomography showing an 8-cm left-sided adrenal tumor with benign () and malignant ( ) differentiated areas. (**b**) Histopathology, left side benign differentiated area (adenoma) ( in **a**) and right side adrenocortical carcinoma ( in **a**). Hematoxylin-Eosin (× 100). (**c**) Malignant differentiated area ( in **a**) with necrosis. Hematoxylin-Eosin (× 100)

All 32 ACC classified tumors showed areas of confluenting tumor cell necrosis (Figs. 2, 3c, 4c, and 5c), irrespective of tumor size (the two smallest tumors measured 3 cm in diameter). One of these small tumors occurred in a 72-year-old male patient with lung metastases diagnosed by biopsy prior to adrenalectomy (Fig. 3). The second patient was an 82-year-old female with suspicious of a left-sided pheochromocytoma (Fig. 4). The adrenal tumor was known for 3 years. Arterial hypertension and elevated catecholamine levels were measured, and tumor size increases by 1 cm within the observation period. Surprisingly, histology showed an ACC potentially arising from an adrenocortical adenoma (Fig. 4b). A similar finding was present in a larger (7 cm in diameter) ACC case containing both adenomatous and carcinomatous areas (Fig. 5).

Confluenting tumor cell necroses were not found in any of the remaining 805 adrenocortical tumors. Thus, sensitivity for predicting metastatic disease by confluenting tumor cell necrosis was 0.75, for specificity 1.00, for positive predictive value 1.00, and for negative predictive value 0.99. The Ki67 proliferation index was > 10% in all but one as ACC classified tumors. The exception was a 7-cm right-sided functioning tumor with a Ki67 index of approximately 5%, which recurred within 1 year both locally and with distant metastases. The 27-year-old female patient died 2 years after the primary surgery. In our series a Ki67 index > 10% was never observed in tumors characterized as ACA.

At primary surgery some ACC cases already showed evidence of malignancy beside the histopathologic findings of the primary tumors. Local invasion with/without venous tumor thrombosis and/or regional lymph node metastases were seen in 8 patients, and distant metastases were already known in 4 other cases. During a mean follow-up of 8.2 years, 24 of 32 patients primarily diagnosed with ACC developed distant metastases (75.0%), whereas none of the patients of the control group had local or distant recurrences. Characteristics of 8 disease-free patients are listed in Table 1.

**Table 1:** Patients with adrenocortical carcinoma and disease-free survival

Patient	Age (years)	Gender	Tumor size (cm)	Site	fu/nonfu	Surgical approach	Ki67 (%)	Disease-free survival (y)
J.C.	49	F	5.5	Right	nonfu	Retrop	10	9.3
W.U.	56	F	9	Left	nonfu	Retrop	12	8.5
S.D.	59	F	12	Right	nonfu	Lapar	25	8.1
S.A.	2	M	4	Left	fu	Retrop	50	5.1
B.L.	26	F	8	Left	fu	Retrop	40	3.4
H.G.	82	F	4	Left	nonfu	Retrop	30	2.5
K.M.	49	M	6	Right	nonfu	Retrop	25	1.0
N.J.	59	M	13.5	Left	fu	Lapar	25	0.9

*F* female, *M* male, *fu* functioning, *nonfu* non-functioning, *retrop* retroperitoneoscopic, *lapar* laparoscopic

The routinely performed fragmentation of larger tumors in MIS cases did not influence histopathologic examination, allowing an unambiguous histological differentiation between benign and malignant neoplasias in all cases investigated.

## Discussion

This study describes a single center experience in adrenocortical tumors overlooking a 20-year period. The surgical team as well as the diagnostic pathologists did not change during this period. Both institutions have an extensive caseload based experience with more than 100 adrenal tumors removed and subsequently histologically examined annually since 2004. At some point the question rose up on how we had differentiated between adrenal neoplasias with favorable or poor clinical course. The main observation was that not a single case of our series was diagnosed false-negative as none of the histologically adrenocortical “adenomas” classified cases ever developed local recurrence or metastatic disease during follow-up. On the other side, the majority of cases (> 70%) diagnosed as ACC proved malignancy unequivocally by means of metastasis development. One limitation of this study is the quality and completeness of follow-up in our group of more than 800 patients. We gained on information by medical reports and phone calls. Autopsies were not included.

During the last decades, scoring systems have become the gold standard to separate benign from malignant adrenocortical tumors. The method proposed by Weiss [4] included nine parameters (atypical nuclei, mitotic rate, atypical mitosis, rate of clear cells, diffuse architecture, venous invasion, sinusoidal invasion, capsule infiltration, confluent necrosis) which counted 1 point each if detected or not. Based on the remarkably low number of overall 43 cases (24 benign, 19 malignant), the author found that all benign tumors were associated with two or less criteria, whereas 18 of 19 specimens with 4 or more parameters recurred or metastasized. In 1985 van Slooten and colleagues published a similar scoring system [5]. After collecting 45 ACC patients and 15 cases with adrenocortical adenomas (ACA), they compared seven histologic parameters (regressive changes, tissue structure, nuclear atypia, nuclear hyperchromasia, structure of nucleoli, mitotic activity, invasion of capsules, and blood vessels) in metastasized (*n* = 42) and non-metastasized (*n* = 18) tumors. By weighting those seven criteria a mathematical threshold was defined. Later, a French group modified the Weiss score after analyzing 24 ACCs and 25 ACAs by weighting the items and extended the system by including the MIB-1 proliferation index [11]. Though they could demonstrate high inter-observer reproducibility among expert pathologists, others had considerable doubts as some Weiss’ features are not easy to evaluate in a standardized manner in daily practice [6]. A common feature of all these reports is that they are based on a rather small number of cases. In contrast, our analysis is based on more than 800 cases and thus a multiple of cases of the previous studies. It should also be emphasized that due to the rarity of ACC, a high number of ACA (clinically proven by the follow-up) must be included in order to confer the analysis a high statistical value.

The main finding in the present study is that all tumors diagnosed as ACC showed areas of confluenting necrosis, whereas this feature was consistently lacking in all cases diagnosed as benign. As this type of necrosis can be easily identified on conventionally stained routine sections (Hematoxylin-Eosin), no further parameters such as nuclei polymorphism, mitotic activity, atypical mitosis, and invasions in veins or tumor capsule have to be analyzed or quantified. Furthermore, immunohistochemistry for determining modern molecular markers such as P53 (tumor suppressor gene TP53), IGF-II (insulin-like growth factor-II), and even Ki67 proliferation index is not necessary or potentially misleading. We would have found at least one false-negative case by relying on a threshold of a Ki67 index > 10%. Thus, the clinical outcome in adrenocortical tumors can be determined by a single histopathologic parameter, thus dramatically simplifying the morphological diagnosis. In our series even very small ACC cases contained unequivocal areas of tumor necroses. Two of these tumors had a size of 3 cm in diameter (Figs. 3 and 4); one of these two cases developed lung metastases as a unequivocal feature of malignancy; the other case demonstrated a potential adenoma-carcinoma sequence, which we consider as a very unusual finding only observed in one further case (Fig. 5). Nevertheless, the parameter “confluenting tumor cell necrosis” in adrenocortical tumors may be used as a screening item to initiate further morphological investigations on effectiveness of adjuvant medical treatment [12]. The latter may allow personalized therapy in those cases.

Beside the described type of confluenting tumor cell necrosis in ACC, spotted areas of necrotic cells or single cell necroses may occur in adrenal tissue. These findings seem to be related to ischemic factors, e.g., in spontaneous intraadrenal bleeding. In such cases malignancy can easily excluded as inflammation and fibrosis dominate the histopathological diagnosis.

Minimally invasive adrenalectomy is surgically performed through ports measuring only 3 to 12 mm in diameter, leading to a frequent discrepancy between surgical incision and tumor size. Most surgeons are thus removing adrenal tumors after widening and dilation of one of the skin incisions in order to follow the demand of their pathologists to receive a (largely) complete encapsulated lesion. Fragmentation of the adrenal tumor prevents or at least hampers the assessment of one of the scoring system criteria, namely, capsular infiltration, whereas all other items are not influenced or altered. Therefore, we strongly recommend the method of tumor fragmentation to avoid unnecessary morbidity (pain, infection) of surgical site; our experience based on more than 800 cases demonstrates that adrenal tumor fragmentation is perfectly compatible with the requirements of the subsequent histological examination.
